# Multi-Mode adhesives performance and success/retention rates in NCCLs restorations: randomised clinical trial one-year report

**DOI:** 10.1080/26415275.2019.1684199

**Published:** 2019-11-06

**Authors:** Patrícia Manarte-Monteiro, Joana Domingues, Liliana Teixeira, Sandra Gavinha, Maria Conceição Manso

**Affiliations:** a Department of Medical Sciences, Faculty of Health Sciences, University Fernando Pessoa, Porto, Portugal;; b Biostatistics, Faculty of Health Sciences, UFP Energy, Environment and Health Research Unit (FP-ENAS), University Fernando Pessoa, Porto, Portugal

**Keywords:** Multi-mode adhesives, adhesion mode, non-carious cervical lesion, randomised clinical trial

## Abstract

**Aim:** Compare clinical performance and success/retention rates of two multi-mode (MM) adhesives, applied in self-etch (SE) or etch-and-rinse (ER) modes, with SE-all-in-one adhesive (SE/SE with enamel etching) in NCCL restorations at one-year follow-up.

**Material and methods:** Prospective, double-blind RCT approved by the University Fernando Pessoa and the National-Clinical-Research-Ethics Committees (CEIC-20150305), ClinicalTrials.gov registered (NCT02698371), in 38 participants with 210 restorations (AdmiraFusion^®^) randomly allocated to six groups (Adhesives_Adhesion mode), each with 35 restorations: G1-Control Futurabond^®^DC_SE; G2-Control Futurabond^®^DC_SE with enamel etching; G3-Futurabond^®^U_ER; G4-Futurabond^®^U_SE; G5-Adhese^®^Universal_ER; G6-Adhese^®^Universal_SE. Restorations evaluated at baseline and one-year by three calibrated examiners (ICC ≥0.952) using FDI criteria and statistical analysis with nonparametric tests (alpha = 0.05).

**Results:** At one-year recall 36 participants, 199 restorations were available for examination; five (2.5%) restorations (G1 *n* = 2; G2, G3, G4 *n* = 1) were lost due to retention (*p* > .05); G1 showed less satisfying marginal adaptation (*p* < .05) than G2 and MM adhesives groups, particularly G6. Overall success rates (*p* > .05) were: 93.9% (G1), 97.0% (G2; G3; G4) and 100.0% (G5; G6).

**Conclusions:** MM adhesives (Futurabond^®^U and Adhese^®^Universal) showed similar and acceptable performance/success rates but also better clinical outputs than the SE-all-in-one adhesive (Futurabond^®^DC), particularly in SE mode. Success and retention rates were similar and not dependent on materials or adhesion modes.

## Introduction

Multi-mode adhesives (MM) are contemporary simplified dental adhesives which can be used either in etch-and-rinse (ER) or self-etch (SE) adhesion approaches. These universal systems allow for the application of the adhesive with phosphoric acid pre-etching, in the total-etch or ER modes, or using selective etching approaches, which enhance enamel bond durability and also provide a simplified SE mode procedure on dentine [1]. The concept behind these adhesives is novel thus only short/medium-term clinical [2–8] and immediate ultra-morphological and bond strength studies have been reported [9–18]. Some laboratory findings have shown that MM adhesive performance is material-dependent [11].

Despite being considered user-friendly [19], this multi-approach enables clinicians to apply adhesives based on the specific clinical situation and operators’ personal preferences [20]. The adjustment of the acidity of MM adhesive solutions and the incorporation of new functional monomers to promote stable clinical performance over time have been the main changes proposed to improve these materials [20,21]. Non-carious cervical lesions (NCCLs) are highly frequent and are normally used in clinical research because they do not present macro-mechanical retention, margins in enamel and/or dentine, and are subject to high stress during masticatory function [22]. The success of NCCL restorations relies mainly on chemical adhesion to the cavity with almost no mechanical retention, particularly with adhesives applied in the ER mode. The impregnation of the dentine substrate by resin monomers and the stability of the bonded interface are of paramount importance to clinical performance [23]. These lesions are considered advantageous when assessing adhesive systems [24,25]. Among other benefits, they offer no mechanical retention and are located mainly in dentine, facilitating evaluation of the resin–dentine bond, which is less stable than the resin–enamel bond [26].

A clinical study of the performance of NCCL restorations evaluated the effects of pre-treatment using phosphoric acid in cases with significant dentine sclerosis. The results showed that only three restorations had failed due to loss of retention within the group where the mild SE adhesive was applied after phosphoric acid pre-treatment of the dentine [27]. Recently, a six-year clinical performance evaluation of ER and SE adhesives revealed that restorations placed on teeth with increased dentine sclerosis were somewhat more likely to lose retention. Although this was not found to be statistically significant, further clinical studies evaluating the clinical performance of restored NCCLs with different degrees of dentine sclerosis are needed [28]. The role of *in vitro* data to predict clinical performance is increasingly recognised, RCTs remain the most rigorous research design for assessing the clinical effectiveness of an intervention. The majority of studies investigating the clinical effectiveness of bonding systems use the longevity of restorations in NCCLs as the outcome [26]. Preliminary data generated from immediate bonding and after thermocycling of dentine specimens bonded with the universal adhesives studied appear to suggest that these adhesives should perform no differently from previous generations of ER adhesives or SE adhesives [29]. The need for clinical reporting regarding the performance of currently marketed MM adhesives led to the design of this study.

The aim of this RCT was thus to compare the clinical performance, success and retention rates of two MM adhesives (applied in SE or ER modes) with an SE-all-in-one adhesive (applied as an SE or SE with enamel etching modes) in NCCL restorations, at one-year recall, using FDI (Word Dental Federation) criteria. The null hypothesis tested at one-year follow-up was: bonding to NCCLs with an SE-all-in-one adhesive (Futurabond^®^DC- FBDC, applied as a SE or SE with enamel etching modes) and MM adhesives (Futurabond^®^U - FBU and Adhese^®^Universal- ADU), applied in SE or ER modes, would result in a non-significant different outcome. To demonstrate this, three null hypotheses (H0) were tested at one-year recall: (H0-1st) similar clinical (aesthetic, functional and biological) performance, (H0-2nd) similar retention rates and (H0-3rd) similar restorations success/acceptance rates.

## Material and methods

### Clinical trial design

**Figure 1.**
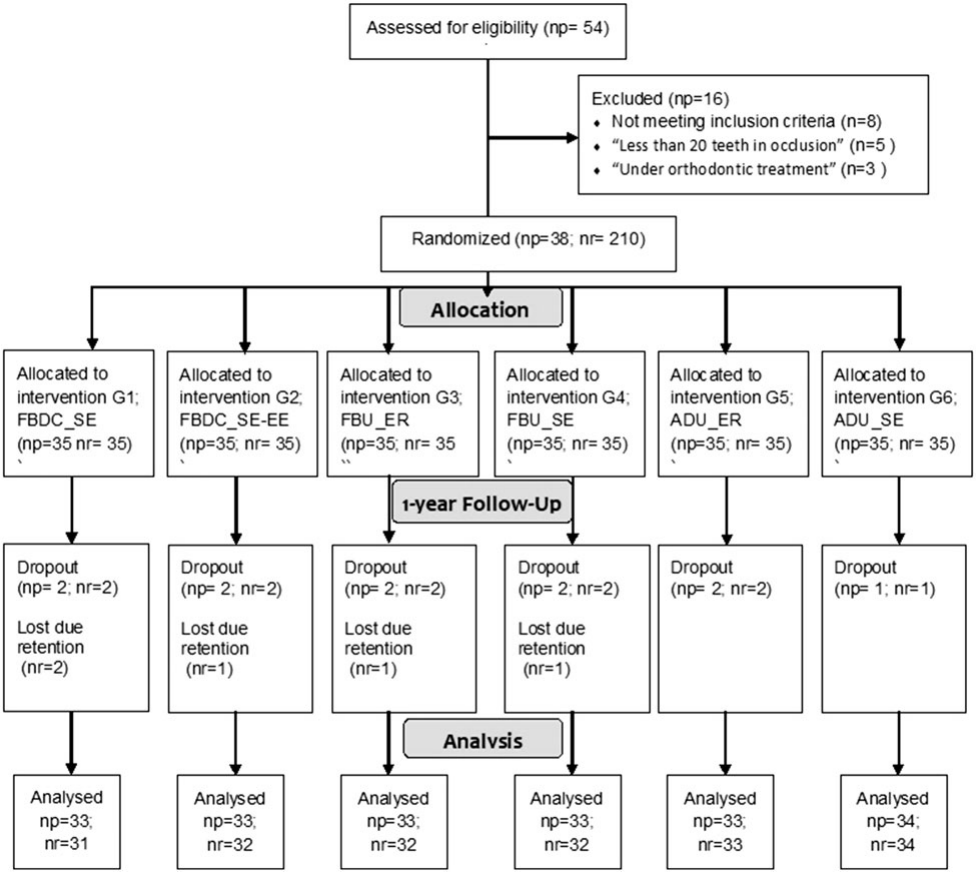
CONSORT Flow Diagram. np: number of patients; nr: number of restorations; SE: self-etch; SE-EE: self-etch with enamel etching; ER: etch-and-rinse; G1-Futurabond^®^DC; (FBDC_SE); G2-FBDC_SE with enamel etching (FBDC_SE-EE); G3-Futurabond^®^U (FBU_ER); G4-(FBU_SE); G5-Adhese^®^Universal (ADU_ER); G6-(ADU_SE).

This prospective, double-blind (patients and examiners) RCT design followed the Consolidated Standards of Reporting Trials (CONSORT) statement [30,31] and the EU directives on good clinical practice for clinical research with medical devices in humans (Figure 1). 210 restorations were randomised to six groups. NCCL restorations were performed between November 2015 and April 2016. The study took place at the Dentistry School Clinic, Faculty of Health Sciences (UFP-FHS), University Fernando Pessoa- Faculty of Health Sciences (UFP-FHS). The baseline clinical observation was conducted 30 days after placement of the NCCL adhesion restorations and the second evaluation at one-year recall.

### Participant selection, inclusion and exclusion criteria

Before the recruitment of participants, the research protocol was approved by the University Fernando Pessoa Ethics Committee, the National Ethics Committee for Clinical Research (CEIC-20150305) and the National Authority of Medicines and Health Products (Infarmed EC/011/2015). This clinical trial was registered at ClinicalTrials.gov (NCT02698371). Voluntary participants aged between 18 to 65 with one to six NCCLs, deeper than 1.5 mm in both enamel and dentine tissues of vital premolar or molar teeth were screened after examination by a single investigator. Patients with a poor medical, psychiatric or pharmacotherapy history, who were pregnant, participating in other ongoing clinical studies, those with allergies or intolerance/adverse reaction to similar products, with less than 20 teeth in occlusion, with severe or chronic periodontal disease or who had undergone periodontal surgery in the past three months, were undergoing orthodontic treatment, suffered from severe bruxism, had poor oral hygiene, with premolars or molar teeth that are anchors for fixed/removal prosthesis, with extreme caries or pulp injuries, or those patients who refused to participate voluntarily in the trial were excluded from the study. Thirty-eight participants signed the informed consent form and were enrolled into this study.

### Resin-based composite, adhesive systems, interventions and restorative procedures

Two hundred and ten NCCL restorations were performed by an experienced and calibrated dentist from the Conservative and Restorative Dentistry Department. The composition and manufacturers of the adhesive systems used are described in Table 1. The adhesives, adhesion mode and application technique are presented in Table 2, according to RCT groups. Participant age and gender description and NCCLs categorised according to tooth type, degree of dentine sclerosis [27], and internal angle shape [2] can be found in Table 3. The number of NCCL restorations per participant varied: three, nine and twenty-six patients received respectively, three, five and six NCCL restorations in one appointment. The allocation of the control (G1, G2) and the study (G3 to G6) groups to each tooth per patient was performed randomly, although ensuring that the group distribution was not repeated in the same patient. Two hundred and ten sealed envelopes were prepared, with 35 sequences of the six study groups, where the order of each of the six groups was randomised. Allocation consisted in opening the envelope on the day of the restorative procedure, with the information (adhesive system/adhesion mode) to be used for that restoration available only to the operator. The operator was not blinded to group assignment during interventions, however, participants were blinded to the group assignment. All operative procedures were performed under anaesthesia (Scandinibsa 3% mepivacaine, Sintra Business Park, Portugal) and relative field isolation, with cotton rolls and retraction cord (Ultrapack #000 or #00, Ultradent, Salt Lake City, UT, USA). After shade guide selection, all NCCLs were cleaned with pumice and water in a rubber cup followed by rinsing and drying. Restorations were placed without an enamel bevel or any mechanical retention. All single dose adhesive systems were applied (Table 2) according to the manufacturers’ instructions and light-cured with a light-emitting diode (LED Unit, Woodpecker; Guilin Woodpecker Medical Instruments Co, Ltd, Welkang Ltd, London) with a power output of 1000 mW/cm^2^ for 20 s. NCCLs were incrementally restored with Admira^®^Fusion Ormofil (Voco, Cuxhaven, Germany). Each increment was light-cured for 20 s, except the last one, which was light-cured for 40 s. After removing the retraction cord, all restorations were finished and polished with polyester discs impregnated with aluminium oxide particles (OptiDisc^®^ medium and extra-fine course; KerrHawe SA; Bioggio, Switzerland) using water spray. Digital photographs of the restorations were taken.

**Table 1.** Information regarding medical devices: manufacturers, Lot# number, composition, adhesives pH value (according to manufactures and safety data sheet).

Medical device (manufacture) Lot#Number	Composition
Futurabond^®^ DC (FBDC) (Voco, Cuxhaven, Germany) Lot# 1532592	Liquid 1. Acidic adhesive monomer*; BIS-GMA (5–10%), 2-HEMA (5–10%); Liquid 2. Ethanol (50–100%); Initiator (2.5–5%) Mixture. organic acids, BIS-GMA, 2-HEMA, TMPTMA, campherchinon, amines (DABE), BHT, catalysts, fluorides and ethanol pH-value 1.5
Futurabond^®^ U (FBU) (Voco, Cuxhaven, Germany) Lot# 1543141	Liquid 1. (2-HEMA) (25–50%); BIS-GMA (25–50%); HEDMA (10–25%); Acidic adhesive monomer (5–10%)*; UDMA (5–10%); catalysts (≤2.5%), silica nanoparticles; Liquid 2. Ethanol (50–100%); Initiator (2.5–5%); catalysts (≤2.5) pH-value 2.3
Vococid^®^ (Voco, Cuxhaven, Germany) Lot#152135	35% orthophosphoric acid
Adhese^®^ Universal (ADU) (Ivoclar Vivadent AG, Liechtenstein) Lot#U35131	Liquid: 2-HEMA (10–<25%); Bis-GMA (10–<25%); ethanol (10–<25%); 1,10-decandiol dimethacrylate (3–<10%); Methacrylated phosphoric acid ester (3–<10%); campherquinone (1–<2.5%); 2-dimethylaminoethyl methacrylate (1–<2.5%); 2-dimethylaminoethyl methacrylate (0.1–<2.5%). pH-value 2.5–3.0
Admira^®^ Fusion (Voco, Cuxhaven, Germany) Lot# (Shade A1, A2, A3, A3,5) 1508270, 150827, 1510508, 1509381	Nano-hybrid ORMOCER^®^s (organically modified ceramics); large and precondensed molecules of an inorganic matrix with a high degree of cross-linking. 84% w/w inorganic fillers. Silicon oxide forms the chemical base, not only for the fillers (nanofillers as well as glass ceramics) but also for the resin matrix.

* Acidic adhesive monomer (10-MDP: 10-methacryloyloxydecyl dihydrogen phosphate according to Voco manufacture); Bis-GMA-Bisphenol A glycidil methacrylate; HEMA-hidroxyethil methacrylate; UDMA- Urethane dimethacrylate.

**Table 2.** Control (G1, G2) and study groups (G3, G4, G5, G6) of NCCL restorations randomised allocation according to adhesives and adhesion mode and, application procedures.

Control and study Groups and NCCL restoration distribution (*n*)	Adhesives system_adhesion mode	Application procedures
G1 (Control group) *n* = 35	FBDC_SE	Mixture Liquid 1 into Liquid 2 (1:1 ratio). Apply and rub this homogeneous mixture to enamel and dentine for 20 seconds; Air-blow for 5 seconds; light cure (1000 mW/cm^2^), for 20 seconds.
G2 (Control group) *n* = 35	FBDC_SE-EE	Apply etchant selectively on enamel and leave for 30 seconds. Thoroughly rinse for 1 minute and gently dry. Dentine surface must slightly remain wet. Mixture Liquid 1 into Liquid 2 (1:1 ratio). Apply and rub this homogeneous mixture to enamel and dentine for 20 seconds; Air-blow for 5 seconds; light cure (1000 mW/cm^2^), for 20 seconds.
G3 *n* = 35	FBU_ER	Apply etchant for 30 seconds on enamel and 15 seconds on dentine; Thoroughly rinse for 1 minute and gently dry. Dentine surface remain with silky matt appearance. Apply and rub adhesive for 20 seconds, and air-blow for 5 seconds; light-cured (1000 mW/cm^2^) for 20 seconds.
G4 *n* = 35	FBU_SE	Apply and rub adhesive for 20 seconds, and air-blow for 5 seconds; light-cured (1000 mW/cm^2^) for 20 seconds.
G5 *n* = 35	ADU_ER	Apply etchant for 30 seconds on enamel and 15 seconds on dentine; Thoroughly rinse for 1 minute and gently dry. Dentine surface remain dry. Scrubbed adhesive for at least 20 seconds; Air-blow to disperse adhesive until a glossy, immobile film layer results; Light-cure (1000 mW/cm^2^) for 20 seconds.
G6 *n* = 35	ADU_SE	Scrubbed adhesive for at least 20 seconds; Air-blow to disperse adhesive until a glossy, immobile film layer results; Light-cure (1000 mW/cm^2^) for 20 seconds.

FBDC: Futurabond^®^ DC; FBU: Futurabond^®^ U; ADU: Adhese® Universal; SE: Self-Etch; SE-EE: SE with enamel etching ER: Etch-and-Rinse.

**Table 3.** Participants demographic features and NCCLs features distribution (*n*, %) of the randomised clinical trial at baseline.

Participant features *N* = 38		
Age		
Me (P_25_–P_75_)	55.5 (41–59)	.999**
min-max	24-63	
Gender		
Female	17 (44.7%)	.999*
Male	21 (55.3%)	
NCCL features *n* = 210		
Tooth type		
Pre-molar tooth	176 (83.8%)	.252*
Molar tooth	34 (16.2%)	
Degree of sclerotic dentine [27]		
Degree 1	146 (69.5%)	.353*
Degree 2	35 (16.7%)	
Degree 3	8 (3.8%)	
Degree 4	21 (10%)	
Cavity geometry (internal shape angle, º) [2]		
Acute (<45º)	84 (40%)	.903*
Acute-to-Right (45º–90º)	60 (28.6%)	
Obtuse (>90º)	66 (31.4%)	

* *p* value according to the Chi-square test or **the Mann-Whitney test.

### Sample size calculation

The sample size calculation was based on rules of thumb, usually considered in research situations where there is little or no information on the outcome, as in this case with the clinical performance of the adhesive systems used in this RCT. Investigators assumed that there was no viable information that would allow for the calculation of sample size based on power analysis. A simpler comparative analysis using a McNemar test (repeated measures) in the six groups, with a total of at least 80 cases (teeth to be restored) was needed in the overall sample. Consequently, at least 35 restorations per group were required for this study. Investigators substantially increased the minimum number stipulated in any of the aforementioned techniques. The outcomes of clinical trials [22,27] using other adhesive systems would suggest that 35 restorations is a feasible number for determining clinical performance-related events in the short to medium-term.

### Clinical evaluation

All restorations were evaluated at baseline (one-month after placement) and at one-year recalls by three calibrated examiners (blinded to study group assignment), using FDI [32] criteria. Intra-examiner (ICC ≥ 0.958) and inter-examiner agreement (ICC ≥ 0.952) were calculated prior to the start-up of the RCT. NCCL restorations were evaluated based on: staining margin (aesthetic property); fractures/retention and, marginal adaptation (functional properties), postoperative (hyper-) sensitivity, recurrence of caries (biological properties), and effect changes (FDI scores). The retention rate was calculated as the percentage of restorations missing due to fractures and retention lost considering the number of restored teeth available for examination, from baseline up to one-year recall. Success rate was defined as the overall percentage of restorations classified with acceptable scores: clinically excellent (EX), good (GO), sufficient/satisfactory (SS) and clinically unsatisfactory, need for repair due to prophylactic reasons (UNS), using the FDI criteria.

### Statistical analysis

Statistical analysis was performed using the IBM^©^ SPSS^©^ Statistics vs. 24 software (IBM Corp. Armonk, NY: IBM Corp.), considering a significance level of 0.05 for all statistical inference. Categorical variables (participants and NCCL features) were described as counts and percentages (n, %) as were the categorical ordinal variables for the aesthetic, functional and biological properties using FDI criteria. The comparison of categorical variables per group was performed using the Chi-Square test, while comparisons of quantitative variables were performed using the Mann-Whitney test (comparison of the median among two groups) and the Kruskal-Wallis test (comparison of median for more than two groups). Pairwise comparison (baseline and one-year) of categorical ordinal variables for the aesthetic, functional and biological properties was completed using the McNemar or the Wilcoxon tests, while cross-sectional comparison between groups at one-year follow-up was performed using the Kruskal–Wallis or the Mann–Whitney tests. The intra and inter-examiner agreement on observations were achieved through the ICC.

## Results

Thirty-eight patients received a total of 210 NCCL restorations distributed across two control (G1, G2) and four (G3 to G6) study groups. No significant differences (Chi-Square test, *p*>.05) were found for all participants and NCCL features (Table 3) and according to RCT groups.

At one-year recall, two (5.3%) participants had dropped out (moved abroad, due to professional reasons); of the 199 NCCL restorations available for evaluation, five (2.5%) were missing due to retention loss (*p* > .05, for all groups and within each group; Table 4). The overall restoration retention rate was 97.5%.

**Table 4.** Research restorations distribution (number) by Control (G1, G2) and Study (G3, G4, G5, G6) Groups, at Baseline and One-year follow-ups, using FDI criteria [32].

FDI Criteria/Score	Restorations (n) by Control (G1, G2) and Study (G3, G4, G5, G6) Groups, at Baseline and 1-year Follow-up											
	G1 FBDC_SE		G2 FBDC_SE-EE		G3 FBU_ER		G4 FBU_SE		G5 ADU_ER		G6 ADU_SE	
	Base	1 y	Base	1 y	Base	1 y	Base	1 y	Base	1 y	Base	1 y
Staining margin												
EX	33	26*	33	29	33	30	33	27*	33	32	34	30*
GO		4*	–	3	–	2	–	4*	–	1	–	4*
SS	**–**	–	–	–	–	–	–	1*	–	–	–	–
UNS	–	1*	–	–	–	–	–	–	–	–	–	–
PO	–	–	–	–	–	–	–	–	–	–	–	–
Fractures and Retention												
EX	33	27*	33	30	33	32	33	31	33	31	34	33
GO	–	3*	–	2	–	–	–	1	–	1	–	1
SS	–	–	–	–	–	–	–	–	–	–	–	–
UNS	–	1*	–	–	–	–	–	–	–	1	–	–
PO	–	2*	–	1	–	1	–	1	–	–	–	–
Marginal Adaptation												
EX	29	18*	31	28	31	28	26	25	30	31	32	28
GO	4	11*	2	2	2	4	7	7	3	1	2	6
SS	–	1*	–	2	–	–	–	–	–	–	–	–
USN	–	1*	–	–	–	–	–	–	–	1	–	–
PO	–	–	–	–	–	–	–	–	–	–	–	–
Postoperative (Hiper-) sensitivity												
EX	33	30	33	32	33	32	33	29	32	29	33	32
GO	–	1	–	–	–	–	–	3	1	4	1	2
Recurrence of Caries												
EX	33	30	33	32	33	32	33	31	33	32	34	33
GO	–	–	–	–	–	–	–	1	–	1	–	1

EX: Clinically excellent/very good; GO: clinically good; SS: clinically sufficient/ satisfactory; UNS: clinically unsatisfactory (repair for prophylactic reasons); PO: clinically poor (replacement necessary); Base: Baseline; 1 y: one-year; *p value (*p* < .05) according to Wilcoxon or McNemar tests, i.e. significant differences from baseline to 1 y follow-up.

Table 4 shows the paired comparison outcome of restoration distribution (number) in the control (G1, G2) and research (G3, G4, G5, G6) groups. At one-year recall, one FBDC_SE (G1) restoration scored as clinically unsatisfactory and four as good (Wilcoxon test; *p* = .016) for staining margin; one FBU_SE (G4) restoration scored satisfactory and four good (*p* = .024), and four ADU_SE (G6) restorations scored as clinically good (*p* = .046). Fractures and retention were recorded as clinically poor (2) [lost retention], unsatisfactory (1), and good (3) in FBDC_SE (G1) (*p* = .026) restorations. One restoration in each FBDC_SE-EE (G2), FBU_ER (G3) and FBU_SE (G4) groups scored as clinically poor (lost retention) and one restoration ADU_ER (G5) was recorded as clinically unsatisfactory (Wilcoxon test, *p* > .05). FBDC_SE (G1) restorations (*p* = .004) showed changes in marginal adaptation, 11 scored as clinically good, one as satisfactory and one as unsatisfactory. Although changes in marginal adaptation occurred over time in all remaining groups, no difference was found within each group (*p* > .05). For postoperative (hyper-) sensitivity parameters, 4 ADU_ER (G5) restorations (one already registered at baseline), 3 FBU_SE (G4) restorations, 2 ADU_SE (G6) restorations (one already registered at baseline) and one FBDC_SE (G1) restoration (within each group, *p* > .05) were classified clinically as good at one-year recall. Recurrence of caries (*p* > .05) scored as clinically good in one restoration from each of the FBU_SE (G4), ADU_ER (G5) and ADU_SE (G6) groups.

At one-year recall, FBDC_SE (G1) restorations revealed a less frequently satisfactory marginal adaptation (Mann-Whitney test, *p* = .039) than those with ADU_SE (G6). Also, FBDC_SE (G1) restorations showed a significantly lower functional performance due to deterioration in marginal adaptation than those with FBU_ER (G3), FBU_SE (G4), ADU_ER (G5) and ADU_SE (G6) (Kruskal-Wallis test; *p* = .003) and those with FBDC_SE-EE (G2) (*p* = .013). No significant difference was found in the clinical performance of FBDC_SE-EE and all the MM adhesives groups. No significant differences were found for biological properties over time within each group (Table 4, Wilcoxon test; *p* > .05).

Overall, the aesthetic, functional and biological property success rates (%) did not differ (*p* > .05) for all groups over a one-year period (Table 5). Success rates were of: 93.9% (FBDC_SE), 97.0% (FBDC_SE-EE and FBU) and 100.0% (ADU). At one-year recall, two (6.1%) FBDC_SE restorations and one (3.0%) FBDC_SE-EE, FBU_ER and FBU_SE lost retention.

**Table 5.** Aesthetic, functional and biological properties success rates by FDI criteria [32] of all groups up to 1-year recall.

Clinical acceptance* (Success rate; %) at one-year evaluation	Control and Research Groups (number of restorations at baseline/one-year)					
	G1	G2	G3	G4	G5	G6
	FBDC_SE	FBDC_SE-EE	FBU_ER	FBU_SE	ADU_ER	ADU_SE
	(33/31)	(33/32)	(33/32)	(33/32)	(33/33)	(34/34)
Aesthetic	100.0	100.0	100.0	100.0	100.0	100.0
Functional	93.9	97.0	97.0	97.0	100.0	100.0
Biological	100.0	100.0	100.0	100.0	100.0	100.0
Overall Success rate (%)	93.9	97.0	97.0	97.0	100.0	100.0

* Acceptance rates (%; percentage) calculated according to *n* = 199 restorations and five restorations missing due to Fractures and retention. All *p* > .05 values according to McNemar test (comparisons between baseline and 1-year evaluation in each group), Kruskal–Wallis test (comparisons of more than two groups) or the Mann–Whitney test (comparisons of two groups).

## Discussion

This research compared the clinical performance, success and retention rates of two MM adhesives (FBU and ADU) applied in SE or ER modes, with an SE-all-in-one adhesive (FBDC) applied using SE or SE with enamel etching (SE-EE) adhesion modes in NCCL restorations at one-year recall, according to FDI criteria. Considering the null hypothesis tested, the first one was rejected because restorations with FBDC_SE showed less satisfying marginal adaptation than those with FBDC_SE-EE and with the MM adhesives, particularly with the ADU_SE. The second and third null hypotheses were not rejected as no difference was found between control and research groups in success and retention rates.

No significant differences were detected for participants and the distribution of NCCL features per group at baseline. A similar number of participants were enrolled in other recently performed clinical trials of the most recent generation of SE [22] and the more recent MM adhesives [2–5,8]. Controversy still remains over whether these versatile adhesives contain technological advances for overcoming the challenges associated with previous generations of adhesives or adhesion modes, since few RCTs were published and very few MM adhesives [2–8] have been tested in NCCL restorations [2–5,8]. Some clinical trials with different evaluation periods (18- to 36-month) tested the same MM adhesive, the Scotchbond^TM^Universal [2–4,8] applied in SE and total-etch modes for NCCL restorations. Other MM adhesives, such as the Xeno^®^ Select and more recently Prime & Bond Elect^®^, have also been tested in RCTs over 6-month [5] and 18-month recalls [8], respectively. In this RCT, two other MM adhesives, the FBU and ADU were assessed. This means that the outputs of this research can be only partially compared with the available scientific data, assessing the products and RCT endpoints.

This clinical research comprised two control groups using FBDC applied in SE mode and with enamel etching (SE-EE). This adhesive was chosen because it is the most recent generation of simplified adhesives from the same manufacture as one of the MM adhesives (FBU) tested. Enamel pre-etching was especially recommended to promote better aesthetic and functional sealing and reduced marginal discolouration/adaptation at the NCCL restoration interface [22,33].

To date, no evidence has been found of clinical research on FBDC, FBU and ADU and these products are available on the market. MM adhesives, similar to previous generations of SE adhesives, differ from one another in many aspects. However their acidity, water content and resin monomer composition are the main features that distinguish them from other adhesives [5]. There is, however, a current trend among manufacturers to continue simplifying single-bottle bonding technology for adhesive procedures, making them faster and if possible, less technique-sensitive [29]. In spite of the poor clinical performance of traditional one-step SE adhesives, the latest generation of all-in-one SE adhesives has performed better, specifically those with ‘mild’ (pH value >1.5) properties [34,35]. Although new adhesives should continue to be compared against an established three-step ER adhesive, since these are supported by the most long-term clinical- and laboratory-based evidence [26], in both recent MM adhesive clinical trials [3,4], only one control group was designed to compare adhesives and adhesion strategies. Additionally, in the RCT conducted by Pena and colleagues, the Clearfil^TM^ SE Bond was selected as control, because it was considered the gold standard for self-etching adhesives and has a clinical performance similar to the three-step ER adhesives [22].

No significant changes in aesthetic, functional and biological performance had occurred at the one-year follow-up within each of the groups FBDC_SE-EE and with FBU and ADU in ER mode.

The staining margin results are partially supported by Lawson and colleagues’ [4] findings in their clinical trial with two-year follow-up, using the USPHS criteria. Although the authors reported that all adhesives tested (Scotchbond^TM^ Universal and Scotchbond^TM^Multi-purpose) showed an increase in marginal discolouration over time, the restorations placed with the MM adhesive in SE mode showed a greater extent of marginal staining than the other material in the 24-month photograph [4]. Therefore, further data at medium-term evaluations and from subsequent clinical recalls must be compared to confirm these results.

Fractures and retention loss occurred significantly over time but only in restorations with FBDC_SE. No difference was detected for this parameter in restorations with FBDC_SE-EE, not even those with FBU and ADU adhesives in SE or ER modes. Lower bonding ability may be related to the chemical bond produced by adhesives with the dental substrates. Although these results would seem to suggest that adhesive systems from the same brand, namely restorations with FBDC (control groups) and FBU, lose retention more frequently than those with ADU, regardless of the SE or ER adhesion modes, no difference was detected for fractures and retention performance between MM adhesives and also, between MM adhesives and the SE-all-in-one adhesive, at one-year follow-up. The results of this RCT support the clinical trial outputs on restoration retention with Scotchbond^TM^Universal which showed four restorations lost at 6-months (one in ER + moist dentine and three in SE mode) and a fifth at the 18^-^month recall (one for selective enamel etching) [2]. Another RCT testing the Scotchbond^TM^Universal reported one restoration failure at the 6-month recall and a second at the 12-month recall in SE mode. In contrast, there was a 100% retention rate when the restorations were applied in ER mode [4]. Similarly, as reported by other authors [2,4], no significant difference was found between retention rates at the 18-month [2] and one-year [4] recall for the Scotchbond^TM^Universal adhesive and adhesion modes, as shown by the outcomes from this RCT, with FBDC, FBU and ADU, in SE and ER modes. Although it is not clear from the FBU safety data sheet that the composition contains 10-MDP (10-methacryloyloxydecyl dihydrogen phosphate), according to the manufacturer this functional monomer is present in the FBDC and FBU [18], and is described as an acidic adhesive monomer. Controversy remains about the use of MMP inhibitors to control the degradation of dentine-resin interfaces [24]. The adhesion-decalcification concept suggests that the aggressive demineralisation of hard tissues by strong acids results in the dissolution of apatite crystallites, decreasing the opportunity to establish chemical bonds between SE adhesives’ functional resin monomers (10-MDP) and apatite crystallites, and the potential for creating nano-layers of calcium precipitates with phosphate resin monomers. However, resin–dentine interfaces created by contemporary MM adhesives containing 10-MDP may not be as immune to degradation as the manufacturers would like [29]. In this RCT all the adhesives, FBDC, FBU and ADU, contain 10-MDP and additionally ADU also contains the functional monomer methacrylated carboxylic acid polymer (MCAP), which may explain some of the current findings at the short-term evaluation. Only FBDC_SE restorations revealed significant deterioration in marginal adaptation at the one-year recall. Additionally, significant functional differences were detected between FBDC, FBU and ADU applied in SE mode. Restorations with FBDC_SE showed less satisfying marginal adaptation than those with FBDC_SE-EE and also those with all the MM adhesives tested, particularly with the ADU_SE. The MM adhesives FBU and ADU showed similar clinical behaviour with regard to the marginal adaptation of the restorations. Previous clinical research reported a similar incidence of non-ideal marginal adaptation and no differences in this parameter for Scotchbond^TM^Universal adhesives applied in selective-etch, SE or total-etch modes, according to Cvar and Ryge evaluation criteria [2,4]. The results of this clinical trial support *in vitro* findings on the effect of acid pre-treatment on the strength of composite resin bonded to enamel and dentine using FBU and Scotchbond^TM^Universal. Although selective enamel etching (SE-EE) of the cavity margins has been recommended to avoid enamel marginal gaps [20,36], *in vitro* results indicate that these adhesives might be a viable option for clinical use, as both FBU and Scotchbond^TM^Universal presented similar bond strengths when used in SE and ER adhesion modes. In general, self-etching adhesive systems, other than MM adhesives, contain acidic monomers responsible for dissolving the smear layer and demineralising dental tissues. Their efficacy depends mostly on the type of monomer used, their pH value, and the application method [18]. The co-variable pH value has been shown to be critical for enamel and dentine bonding, although pH alone does not directly correspond to bond strength and/or interface morphology [27]. According to the manufacturer, the pH of FBDC is 1.5 and that of, FBU 2.3 and of ADU 2.5. Therefore, when considering the SE adhesion mode, restorations with FBDC should have had better marginal adaptation than the MM adhesives, FBU and ADU, as the lower the pH of the adhesive, the better the etching on enamel. Instead, FBU and ADU with a higher pH performed better in terms of marginal adaptation. This short-term output would seem to suggest that clinical performance may depend not only on the type/composition but also on the concentration of the monomer formulation and that this is product-dependent [35]. The functional acidic monomer concentration and composition, particularly those that regulate hydrophilicity and water content, such as the HEMA (2-hydroxyethyl methacrylate), may explain the change in performance of some brands of MM adhesives. One-step SE adhesives are highly hydrophilic (regulated by HEMA) so they attract water from dentine tubules, which may increase the potential for degradation, as water sorption of adhesive resins is proportional to their hydrophilic characteristics. Increasing the water concentration dilutes the acidic monomer concentration and may decrease their bonding effectiveness and their mechanical/functional properties [36]. As FBU and ADU both contain HEMA a poorer marginal adaptation performance could be expected [37].

In this RCT, no differences were found in the biological properties over the time within each group or between the adhesives or adhesive modes. However, restorations with ADU (in the SE or ER mode) and FBU_SE more frequently revealed some postoperative sensitivity at the one-year follow-up, receiving a score of clinically good. Further long-term evaluation is needed to investigate the possible influence of MM adhesives on postoperative sensitivity and recurrence of caries.

Using the clinical index introduced by Heintze (2009) [38] and Heintze (2010) [39] and colleagues, we obtained an overall *in vivo* success rate of 96.5% for FBDC_SE, 98.3% for FBDC_SE-EE and FBU and, 100.0% for ADU. These values show extremely low deterioration, which supports some initial values (12-months) used in the meta-analysis and above the overall average out of all the studies for that time point (average portrayed using a big dot in Figure 1, graph d) to model deterioration over time (one to three years) [39].

This RCT research protocol included some of the main topics identified by authors [2–4,22,27,28] such as, the clinical evaluation by FDI criteria (sensitive criteria for short-term evaluations), three calibrated independent examiners, participants and NCCL features.

In conclusion, NCCLs restored using FBU and ADU showed similar aesthetic, functional and biological performance and performed clinically better than NCCLs restored with FBDC_SE. Restorations with FBU and ADU applied in SE mode revealed less frequent changes in marginal adaptation than those with FBDC_SE. Retention and success rates were similar at one-year recall and did not depend on adhesion modes (SE and ER) nor adhesive systems (FBDC, FBU and ADU).
